# Ecological niche adaptation of *Salmonella* Typhimurium U288 is associated with altered pathogenicity and reduced zoonotic potential

**DOI:** 10.1038/s42003-021-02013-4

**Published:** 2021-04-23

**Authors:** Mark Kirkwood, Prerna Vohra, Matt Bawn, Gaëtan Thilliez, Hannah Pye, Jennifer Tanner, Cosmin Chintoan-Uta, Priscilla Branchu, Liljana Petrovska, Timothy Dallman, Neil Hall, Mark P. Stevens, Robert A. Kingsley

**Affiliations:** 1 Quadram Institute Biosciences, Norwich Research Park, Norwich, NR4 7UQ UK; 2 Roslin Institute, Edinburgh, EH25 9RG UK; 3 Earlham Institute, Norwich Research Park, Norwich, NR4 7UZ UK; 4 University of East Anglia, Norwich, NR4 7TJ UK; 5 Animal and Plant Health Agency, Addlestone, KT15 3NB UK; 6 Gastrointestinal Bacteria Reference Unit, National Infection Service, Public Health England, London, NW9 5EQ UK; 7 Present Address: Institute for Immunology and Infection Research, School of Biological Sciences, University of Edinburgh, Edinburgh, EH9 3FL UK

**Keywords:** Pathogens, Phylogenetics

## Abstract

The emergence of new bacterial pathogens is a continuing challenge for agriculture and food safety. *Salmonella* Typhimurium is a major cause of foodborne illness worldwide, with pigs a major zoonotic reservoir. Two phylogenetically distinct variants, U288 and ST34, emerged in UK pigs around the same time but present different risk to food safety. Here we show using genomic epidemiology that ST34 accounts for over half of all *S*. Typhimurium infections in people while U288 less than 2%. That the U288 clade evolved in the recent past by acquiring AMR genes, indels in the virulence plasmid pU288-1, and accumulation of loss-of-function polymorphisms in coding sequences. U288 replicates more slowly and is more sensitive to desiccation than ST34 isolates and exhibited distinct pathogenicity in the murine model of colitis and in pigs. U288 infection was more disseminated in the lymph nodes while ST34 were recovered in greater numbers in the intestinal contents. These data are consistent with the evolution of *S*. Typhimurium U288 adaptation to pigs that may determine their reduced zoonotic potential.

## Abstract (web summary)

Mark Kirkwood et al. take an epidemiological, genomic, phylogenetic, and experimental approach to assess the evolutionary origins and virulence of the U288 and ST34 isolates of *Salmonella enterica* in affecting livestock and potentially humans. Their results indicate that the molecular evolution of these isolates is associated with altered pathogenicity in pigs and reduced zoonotic potential of U288.

## Introduction

Emergence of infectious diseases presents new challenges for the management of human and livestock health, with substantial human and economic costs through morbidity and mortality, and lost productivity in agriculture. The emergence of 335 human infectious diseases between 1945 and 2004 was dominated by zoonoses of bacterial aetiological agents.^1^ A total of 10 of the 335 emergent infectious diseases during this period were *Salmonella enterica* and several more have been reported since, including *S. enterica* serotype Typhimurium (*S*. Typhimurium) ST313 associated with invasive non-typhoidal *Salmonella* (iNTS) disease in sub-Saharan Africa, and extensively drug resistant (XDR) *S*. Typhi.^2–4^ *Salmonella* was estimated to have caused around 87 million human infections resulting in approximately 1.2 million deaths globally in the year 2010. Non-typhoidal *Salmonella* alone has the greatest impact on health with 4 million disability adjusted life years lost, the greatest burden on human health among foodborne diseases.^5^ Pigs are one of the major zoonotic reservoirs, with 10–20% of human salmonellosis in Europe attributable to them.^6,7^ An understanding of the evolutionary processes leading to the emergence of new infectious diseases has the potential to improve pathogen diagnostics and surveillance, and guide policy and interventions aimed at decreasing the burden of human and animal infection.

The genus *Salmonella* consists of over 2500 different serovars that have diverse host ranges, pathogenicity and risk to human health. One of these serovars, *S*. Typhimurium (including monophasic variants), has consistently been a dominant serovar in pigs globally, and currently accounts for around two thirds of isolates in the UK.^8,9^ Despite the ostensibly stable prevalence of *S*. Typhimurium in pig populations over time, the epidemiological record indicates a dynamic process where distinct variants, identified by phage typing, increase and decrease in prevalence over time.^8^ Since the middle of the 20^th^ century in Europe the dominant phage types were definitive type 9 (DT9), DT204, DT104 and most recently DT193 that is a monophasic *S*. Typhimurium (*S*. 1,4,[5],12:i:-) with sequence type 34 (ST34).^10,11^ At their peak incidence, each accounted for over half of all human isolates of *S*. Typhimurium. Phage typing has been useful for surveillance and outbreak detection, but only provides limited information about the relationship of the *Salmonella* isolates due to their polyphyletic nature and potential for rapid changes in phage type as a result of mutations and horizontal gene transfer.^12^ Nonetheless, sub-genomic and whole genome sequence analysis confirmed that the emergence of new phage types over time does represent the emergence of distinct clonal groups.^13,14^ The drivers of their emergence and the consequences for human and animal health are largely unknown.

Since around the year 2003, *S*. Typhimurium isolates of U288 and DT193 have dominated UK pigs.^8^ U288 appeared in UK pig populations around 2003 followed around the year 2006 by monophasic *S*. Typhimurium (*S*. 1,4,[5],12:i:-) ST34 rapidly emerging in pig populations around the world.^8,15,16^ U288 and ST34 co-existed in the UK pig population and together accounted for around 80% of isolates.^17^ Despite, approximately half of all pork consumed in the UK being from UK pig herds,^18^ since its emergence U288 have rarely been isolated from human infections in the UK.^17^ In contrast, by the year 2013 in the UK over half of all *S*. Typhimurium infections in the UK were due to ST34, reflecting its capacity to be transmitted through the food chain and cause human infections.^19,20^ U288 is not a definitive type and the designation is not widely adopted outside of the UK. Consequently, the prevalence of U288 outside of the UK is unclear. However, we previously detected U288 in pigs in Ireland,^21^ a study reported that it was widespread in Danish Pig herds,^22^ and was present in Italy.^23^

A baseline survey reported prevalence of 21.2% and 30.5% in mesenteric lymph nodes and caecal contents for UK slaughter pigs in studies from 2007 to 2013, respectively.^24,25^ It is believed that contamination of pig carcasses with faeces and gut contents at slaughter, and the ability of *Salmonella* to spread from the gut to other organs, results in contamination of meat products that enter the food chain and pose a risk to humans if improperly handled or cooked. However, the relative risk from contamination of meat by gut contents during slaughter or from tissue colonised by *Salmonella* prior to slaughter is not known and could be affected by differences in pathogenesis depending on the genotype of *Salmonella* involved. Survival of *Salmonella* in food depends upon adaptive response to environmental stresses including osmotic stress from biocides and desiccation, antimicrobial activity of preservatives and fluctuating temperatures during storage or cooking. In order to cause disease, *Salmonella* may also need to replicate in food to achieve a population size able to overcome the colonisation resistance of the host.

Multiple pathovariants of *S*. Typhimurium are thought to have evolved from a broad host range ancestor resulting in distinct host range, outcome of infection and risk to food safety,^12,26,27^ similar to that observed for distinct serovars.^28^ An understanding of the molecular basis of risk to food safety of *S*. Typhimurium pathovariants is critical to improve assessment of risk and devise intervention strategies aimed at decreasing *Salmonella* presence in food. Furthermore, the identification of genomic signatures of zoonotic risk of *Salmonella* has the potential to further improve source attribution in outbreak investigations, as recently shown using machine learning approaches.^12,29,30^ We therefore investigated the population structure of *S*. Typhimurium U288 and the genomic evolution accompanying the clonal expansion of U288 by analysis of whole genome sequences. The objective was to identify representative isolates of the U288 epidemic clade and compare their interaction with the environment and the pig host to gain insight into the phenotypic consequences of their distinct evolutionary trajectories.

## Results

### *S*. Typhimurium U288 and monophasic *S*. Typhimurium ST34 exhibit distinct host range

**Fig. 1:**
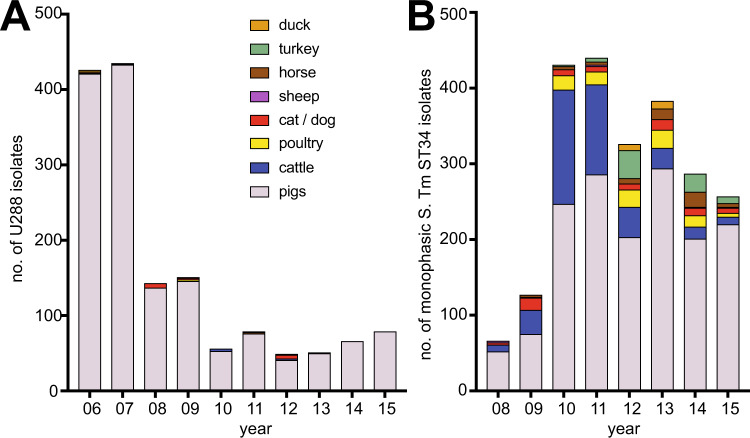
Animal species source of monophasic *S*. Typhimurium U288 and *S*. Typhimurium ST34. Stacked bar chart indicating the animal source (see colour key inset) of *S*. Typhimurium U288 (**A**) and monophasic S. Typhimurium ST34 (**B**) isolated in England and Wales by Animal and Plant Health Agency 2006–2015.

The epidemiological record indicates that *S*. Typhimurium U288 was first reported in pigs in the UK around the year 2000 and thereafter became the dominant phage-type isolated for much of the following decade.^8^ Monophasic *S*. Typhimurium ST34 emerged around seven years later in UK pigs, and these two variants have co-existed in pig populations since. Retrospective analysis of the frequency of U288 and monophasic *S*. Typhimurium ST34 isolated from animals in the UK by the Animal and Plant Health Agency (APHA) between 2006 and 2015, revealed distinct host ranges (Fig. 1). During this period, a total of 1535 and 2315 isolates of *S*. Typhimurium U288 and monophasic *S*. Typhimurium, were reported by APHA from animals in the UK, respectively. *S*. Typhimurium U288 was almost exclusively isolated from pigs, while in contrast, monophasic *S*. Typhimurium, although predominantly isolated from pigs,^31^ was also isolated from multiple host species including cattle and poultry populations (Fig. 1). Emergence of ST34 coincided with a decrease in number of U288 isolates, although both variants were present throughout this time.

### The *S*. Typhimurium U288 and ST34 isolates form distinct phylogroups

To investigate the phylogenetic relationship of *S*. Typhimurium U288 isolates, we first constructed a maximum likelihood tree using variation in the recombination-purged core-genome sequence of 1826 *S*. Typhimurium isolates from human clinical infections in England and Wales between April 2014 and December 2015 for which both whole genome sequence and the phage-type data were available. From all isolates 24 (1.4%) were U288 and of these, 20 were present in a distinct clonal group, composed of 33 isolates in total (henceforth referred to as the U288 clade, Supplementary Fig. 1). Four U288 were in a distinct outlier clade. The remaining 13 isolates within the predominantly U288 clade were reported as phage types DT193 (5 isolates), U311 (3 isolates), U302 (1 isolate), or reacted did not conform (RDNC, 4 isolates) and may be mis-typed or naturally occurring phage-type variants. The main U288 clade was closely related to 13 human clinical isolates, of various phage types, but predominantly U311; none were U288.

**Fig. 2:**
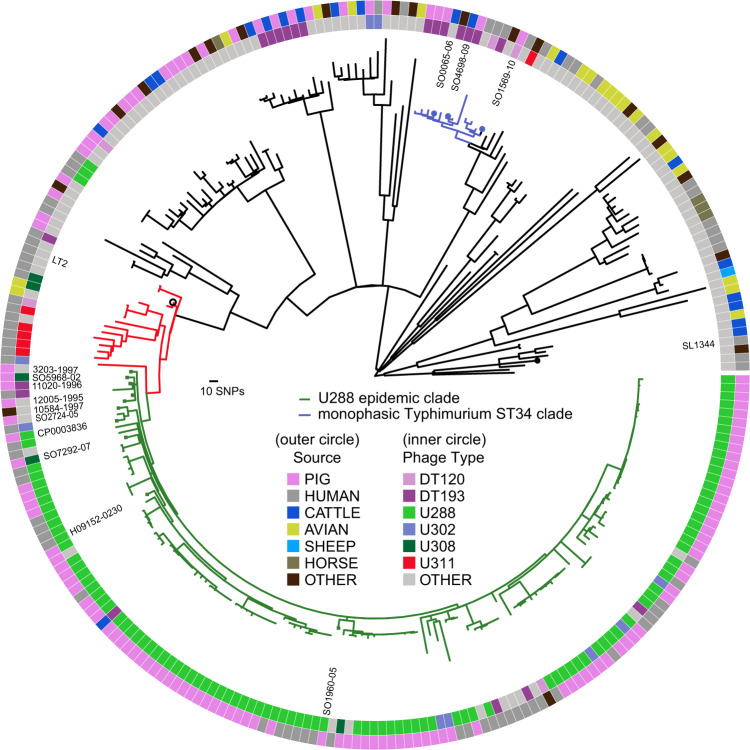
Phylogenetic relationship of *S*. Typhimurium U288 and *S*. 4,[5],12:i:- epidemic clades in the context of *S*. Typhimurium diversity. Mid-point rooted maximum likelihood phylogenetic tree constructed using 7189 SNPs from the recombination-purged core genome sequence of 262 *S*. Typhimurium isolates: 79 U288 strains isolated from animals in the UK in the years 2014 and 2015, 36 human clinical strains from the main U288 clade isolated from 2014 and 2015, a U288 isolate (CP0003836), reported previously from Denmark in 2016,^32^ 131 isolates from the UK that represented diverse phage types,^12^ and 15 closely related but non-U288-clade isolates. The source of isolate (outer circle) and phage-type of isolate (inner circle) are specified by the fill colour as indicated in the key. *S*. Typhimurium U288 (green lineages), closely related to U288 (red lineages) and monophasic *S*. Typhimurium ST34 (blue lineages) are indicated.

To investigate the relationship of contemporaneous *S*. Typhimurium U288 in the UK pig population and human clinical isolates, we determined the whole genome sequence of 79 *S*. Typhimurium U288 strains isolated from animals in the UK in the years 2014 and 2015 as part of APHA surveillance. To place these in the phylogenetic context of *S*. Typhimurium, we included 128 isolates from the UK that represented diverse phage types,^12^ that included 12 isolates from the current monophasic *S*. Typhimurium ST34 epidemic.^27^ We also included the 36 human clinical strains from the main U288 clade isolated from 2014 and 2015, 3 U288 isolates outside of the main clade, 15 closely related but non-U288-clade isolates, and a U288 isolate (CP0003836), reported previously from Denmark in 2016.^32^ The phylogenetic structure of *S*. Typhimurium was consistent with that described previously,^12^ with a number of deeply rooted lineages, some of which exhibited evidence of clonal expansion at terminal branches (Fig. 2). All *S*. Typhimurium U288 isolates from pigs were present in a single phylogenetic clade together with the 33 isolates from human clinical infections (U288 clade, green lineages, Fig. 2). The U288 clade was closely related to sixteen *S*. Typhimurium isolates of various other phage types but none were phage type U288. Most of these were isolated from human clinical infections, and two from avian hosts (Fig. 2). Of note, *S*. Typhimurium strain ATCC700720 (LT2) differed by fewer than 5 SNPs from the common ancestor of the U288 clade and the 13 related non-U288 strains. *S*. Typhimurium strain ATCC700720 (LT2) was originally isolated from a human clinical infection at Stoke Mandeville hospital, London in 1948, and subsequently has been used for studying the genetics of *Salmonella* worldwide.^33^ The three U288 isolates from human clinical infections in the minor U288 clade clustered together with isolates of other non-U288 phage types.

We next estimated the relative contribution of the *S*. Typhimurium U288 clade isolates and monophasic *S*. Typhimurium ST34 clade isolates to human clinical infections in the UK between April 2014 and December 2015. Of 1826 *S*. Typhimurium isolated in this period 33 (1.8%) were from the U288 clade. In contrast, 894 isolates (49%) were from the monophasic *S*. Typhimurium ST34 clade.

To estimate the global distribution of U288 clade strains isolated outside of the UK we examined 34,487 *S*. Typhimurium whole genome sequences in the Enterobase database. We identified a hierarchical cluster (HC20-201) composed of 455 genomes, of which 345 reported the country of origin, that contained all strains reported as U288. This cluster was consistent with the main U288 phylogenetic cluster^34^ (Supplementary Fig. 2). HC20-201 also contained 254 genomes from strains isolated in the UK and 91 from France, Denmark, Italy, Germany, Ireland, Austria, or the US. Significantly, the proportion of all *S*. Typhimurium in Enterobase that were HC20-201 in the European countries ranged from 0.15% to 7.6%, similar to the 2.1% observed for UK *S*. Typhimurium isolates in Enterobase.

### Distinct prophage repertoire, plasmid content, and genome degradation of *S*. Typhimurium U288 strain S01960-05 and monophasic *S*. Typhimurium ST34 strain S04698-09

**Fig. 3:**
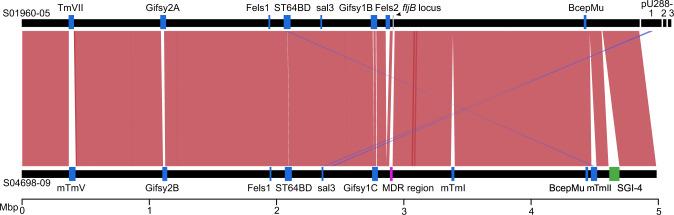
Alignment of *S*. Typhimurium U288 strain S01960-05 and ST34 strain S04698-09 chromosome sequence. Genomes are indicated by horizontal black lines including pU288-1, pU288-2 and pU288-3. Scale bar indicates Mbp of nucleotide sequence. Shaded segments connecting the genomes indicate colinear (red) or reverse and complement (blue) regions with nucleotide sequence identity >90%. Notable features highlighted by coloured boxes and labels for prophage (blue), the SGI-4 integrative conjugative element (green), *fljB* locus (grey) and a resistance region (red).

To compare the whole genome sequence of U288 strain S01960-05^12^ and ST34 S04698-09^14^ the closed genomes were aligned. The genomes exhibited overall synteny, that was interrupted by seven insertions or deletions (indels) greater than 1 kb due to distinct prophage occupancy, recombination within shared prophage in one or other genome, the presence of a resistance region encoding multidrug resistance, and an integrative conjugative element (SGI-4) in strain S04698-09 (Fig. 3). Both genomes had Gifsy1, Gifsy2, ST64B, similar partial sequence of Fels-1, and remnant prophages SJ46 and BCepMu. The *thrW* locus was variably occupied by either the mTmV prophage in monophasic Typhimurium ST34 strain S04698-09 that carries the *sopE* gene,^14^ or ST104 in U288 strain S01960-05, a prophage previously described in *S*. Typhimurium DT104 strain NCTC13384.^13^ U288 strain S01960-05 had a complete Fels-2 prophage which was absent from S04698-09. The S04698-09 genome also harboured two additional prophages related to HP1 and SJ46, that were absent from U288 strain S01960-05.

*S*. Typhimurium U288 strain S01960-05 contained three plasmids reported previously in another U288 strain from Denmark, pU288-1, pU288-2 and pU288-3.^32,35^ In contrast, no plasmids were present in monophasic *S*. Typhimurium ST34 strain S04698-09. pU288-1 is similar to the virulence plasmid pSLT, an IncF plasmid present in many *S*. Typhimurium strains.^35^ Additional sequence in pU288-1 but absent in pSLT included an integron with AMR genes including, *dfrA12, aadA2, cmlA, aadA1 and sul3*, and the *bla*_TEM_ gene associated with an IS26-like element. The IncQ1 plasmid pU288-2 encoded additional AMR genes *sul2*, *strA*, *strB*, *tetA*(A) and *cat*.

A notable difference in coding capacity affecting the core genome of the S01960-05 and S04698-09 strains resulted from hypothetically disrupted coding sequences (HDCS) due to the introduction of a premature nonsense codon from small insertions or deletions (indels) resulting in a frameshift or a single nucleotide polymorphisms (SNP) giving rise to a new nonsense codon. The monophasic *S*. Typhimurium ST34 strain S04698-09 genome contained three HDCS outside of prophage, with reference to *S*. Typhimurium SL1344. In contrast, *S*. Typhimurium U288 strain S01960-05 contained 19 HDCS outside of prophage (Table 1). Fifteen U288 HDCS encoded hypothetical proteins of unknown function (*ygbE*, *yciW*, SL2283, *yfbK*, SL2330, *yhbE*, SL0337, *ybaO*, SL1627, *yfbB*, *yqaA*, SL1627, SL0337, SL2330 and SL2283), while eleven had predicted functions based on sequence similarity with proteins of known function (*assT5*, *assT3*, *dtpB*, *hutU*, *cutF*, *oadA*, *pncA*, *sadA*, *tsr*, *oatA*, and *rocR*).

**Table 1:** Hypothetically disrupted coding sequence (HDCS) in U288 strain S01960-05 with reference to strain S04698-09.

Polymorphism	SL1344 locus	Gene	Product	Fitness score*	no. mutants
del	0242	*nlpE*/*cutF*	Copper homeostasis protein precursor/lipoprotein precursor	+1.26	2
ins	0337	NA	Hypothetical periplasmic protein	−1.5	1
ins	0453	*ybaO*	Hypothetical transcriptional regulator	—	0
ins	0744	*oadA2*	Oxaloacetate decarboxylase alpha chain	−0.32	3
ins	0767	*hutU*	Urocanate hydratase	+1.19	1
ins	0987	NA	Hypothetical host specificity protein (Gifsy-2)	—	5
ins	1228	*pncA*	Nicotinamidase/pyrazinamidase	—	0
del	1627	NA	Conserved hypothetical protein	**−2.96**	1
nsSNP	1632	*yciW*	Conserved hypothetical protein	−0.87	1
ins	2208	*oafA*/*oatA*	O-antigen acetylase/ O-acetyltransferase	**−1.52**	10
del	2274	*menE*	O-succinylbenzoic acid-CoA ligase	−0.65	4
ins	2277	*yfbB*	Conserved hypothetical protein	—	
del	2282	*elaC*	Ribonuclease Z	**−4.03**	1
del	2283	*NA*	Hypothetical receptor/regulator protein	−0.13	0
del	2284	*yfbK*	Lipoprotein	+0.27	1
del	2285	*nuoN*	NADH-quinone oxidoreductase subunit N	—	0
ins	2330	*rocR*	Hypothetical transcriptional regulator/Arginine utilization protein	+0.20	2
del	2661	*bapA*	Beta-peptidyl aminopeptidase	+1.13	19
ins	2804	*yqaA*	Putative membrane protein	+1.08	1
del	2911	*ygbE*	Inner membrane protein	+2.43	1
ins	3165	*assT3*	Probable arylsulfate sulfotransferase	**−5.76**	2
ins	3274	*yhbE*	Hypothetical membrane protein/ inner membrane transporter	−0.77	1
ins	3557	*dtpB*	Dipeptide and tripeptide permease	—	0
nsSNP	3656	*sadA*	Autotransporter/adhesin	**−0.08**	8
del	3760	*NA*	Transcriptional regulator	+0.66	1
nsSNP	3934	*assT5*	Probable arylsulfatase sulfotransferase	**−2.05**	4
ins	4464	*tsr*	Methyl-accepting chemotaxis protein	—	0

The type of sequence polymorphism leading to disruption is indicated as insertion (ins), deletion (del), or non-sense codon (nsSNP) with reference to sequence (S04698-09). The corresponding locus tag in reference strain SL1344 and the predicted function is indicated. * Fitness score was derived from the screening of *S*. Typhimurium strain ST4/74 mutants by oral inoculation of pigs and recovery from the colon using transposon-directed insertion site sequencing.^47^ The fitness score is the log_2_-fold change in the number of sequence reads across the boundaries of a transposon insertion in the gene between the input and output pools, after normalisation to account for variations in the total number of reads obtained for each sample. Zero indicates no change in relative abundance, positive values indicate mutants that are more abundant and negative values less abundant in the output pool recovered from the colon. Where multiple mutants for a gene were screened, the mean score is presented with the number of mutants indicated. Where the fitness score for one or more of the mutants in the output pool were reported to be significantly different from the input pool these are indicated (bold type).

### The pangenome of U288 and ST34

Analysis of the pangenome revealed a similar-sized core and accessory genome, with 3962 and 4056 core gene families, out of pangenomes of 5501 and 5578 gene families, for U288 and ST34, respectively (Supplementary Figure 3). The key differences in the genome sequence of reference strains S01960-05 and S04698-09 included the presence of SGI-4 in ST34, and the presence of pU288-1 (pSLT related) in U288, were general lineage-specific characteristics as they are present in most strains in each case. Lineage-specific differences in the accessory genome were due to distinct prophage repertoires, the presence of SGI-4 in ST34, and the presence of pU288-1 (pSLT related) in U288. The prophage repertoire was somewhat variable within the ST34 lineage and relatively invariant in U288, while plasmid sequence, including pU288-1 was highly variable in U288 and only occasional acquisition of small plasmids in ST34. Notably, U288 lacked lineage-specific genes present on the chromosome, with the exception of prophage.

### The *S*. Typhimurium U288 clade evolved from an *S*. Typhimurium LT2-like common ancestor by genome degradation and acquisition of AMR genes

We next investigated the genotypic variation of AMR genes, plasmid replicons and plasmid sequence within the U288 clade to infer the evolutionary events associated with the emergence of the U288 clade. To this end we identified genes by mapping and local assembly of short-read sequence data of 148 U288 clade isolates and related *S*. Typhimurium isolates to databases of plasmid replicons, AMR genes and allelic variants associated with HDCS.

**Fig. 4:**
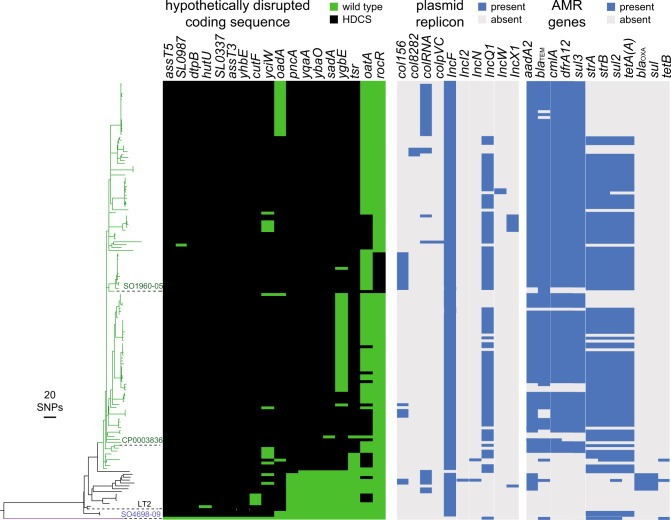
Phylogenetic relationship and genotypic variation in selected genes of *S*. Typhimurium U288 and monophasic *S*. Typhimurium ST34 strain S04698-09. Mid-point rooted maximum likelihood tree based on 1859 SNPs in the recombination-purged variation in the core genome with reference to *S*. Typhimurium strain SL1344. Lineages associated with the U288 (green), closely related strains (black) and ST34 S04698-09 (purple) are indicated. Scale bar indicates the estimated number of SNPs based on the genetic distance as a fraction of total SNPs. This collection comprised 134 *S*. Typhimurium U288 isolates from animals and human infections in the UK placed in the context of 16 *S*. Typhimurium isolates from animals and human that were closely related but outside of the main U288 clade. Monophasic *S*. Typhimurium strain S04698-09 is included as an outgroup. The presence of allelic variants associated with HDCS, plasmid replicons and resistance genes are indicated by bars colour coded as indicated in the key (inset).

The IncF replicon was present in all isolates from the U288 clade and closely related isolates, consistent with the presence of all or part of the pSLT plasmid-associated sequence (Fig. 4). Deletions of large parts of the pSLT-associated sequence were evident in the U288 clade isolates (Supplementary Fig. 3C). Furthermore, in 58 of 133 U288 clade isolates, deletions affected two or more of the *spv* genes, previously implicated in virulence in the mouse model of infection (Supplementary Fig. 3C)^36^.

The pattern of AMR gene presence was consistent with acquisition in two distinct evolutionary events. First, an IncQ1 plasmid (pU288-2) was acquired concurrent with initial clonal expansion of the clade, followed by subsequent acquisition of a transposon on the pSLT-like plasmid, pU288-1 (Fig. 4). The IncQ1 replicon of the pU288-2 plasmid was present in 97 of 133 U288 clade isolates, including a cluster of six most deeply rooted isolates in the U288 clade, and was associated with the *strA, strB, sul2* and *tetA*(A) genes, encoding resistance to streptomycin, sulphonamide and tetracycline antibiotics. AMR genes *cmlA*, *sul3*, *dfrA12, aadA2* and *bla*_TEM_ that confer resistance to chloramphenicol, sulphonamides, trimethoprim, aminoglycoside and β-lactam antibiotics respectively were present on pU288-1 in all but 16 U288 clade isolates. These AMR genes were absent from six of the most deeply rooted isolates in the *S*. Typhimurium U288 clade.

Investigation of the distribution of the 19 HDCS identified in *S*. Typhimurium U288 strain S01960-05 and within diverse *S*. Typhimurium strains in the context of their phylogenetic relationship indicated their sequential acquisition during the evolution of the U288 clade (Fig. 4). Of the 19 HDCS, six (SL0337, *yhbE*, *cutF*, *yciW* and *oadA*) were also present in the genome sequence of closely related isolates including LT2, and four (*assT5*, SL0987, *dtpB* and *hutU*) were present in isolates from two relatively distinctly related clades. Two additional HDCS in *S*. Typhimurium U288 strain S01960-05, were either sporadically present as HDCS throughout the *S*. Typhimurium collection (*oatA* and *rocR*), or only present as HDCS in strain S01960-05 and 14 closely related isolates. Six HDCS (*pncA*, *yqaA*, *ybaO*, *sadA*, *ygbE* and *tsr*) were present in most U288 clade isolates, although a wild type allele of one of these (*ygbE*) was present in a subclade containing 35 isolates, and two other U288 isolates, suggesting that these may have subsequently reverted.

### AMR gene acquisition and genome degradation preceded the U288 epidemic clonal expansion

**Fig. 5:**
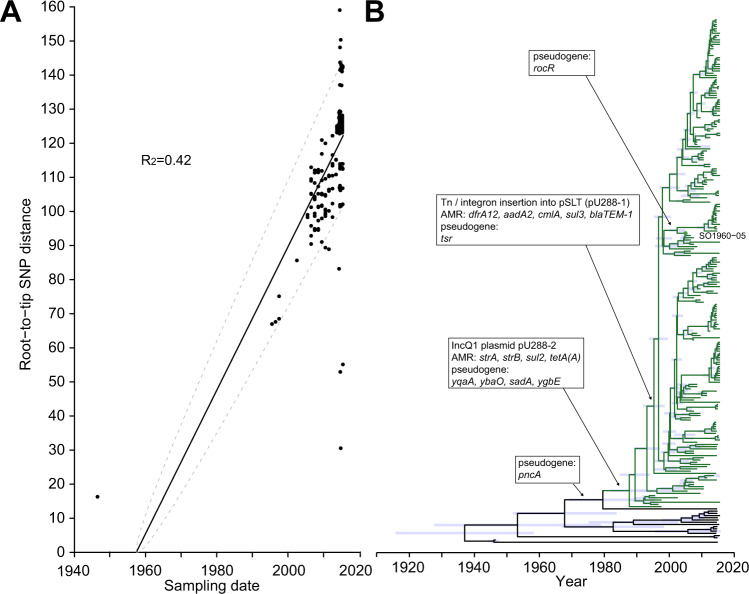
Time-scaled phylogenetic analysis of the emergence of the U288 epidemic clade. Analysis of 234 U288 clade and closely related strains isolated between 1947 and 2015 calculated using maximum likelihood estimation based on recombination-purged variation in the core genome sequence. (**A**) Linear regression of root-to-tip SNPs with a slope of 2.05 SNPs per year, and (**B**) estimated time-scaled phylogeny, manually rooted using monophasic *S*. Typhimurium ST34 strain S04698-09 as an outgroup that was subsequently removed before further analysis. Blue bars at nodes indicate 95% CI of dated nodes, green lineages indicate the U288 epidemic clade, and black lineages indicate lineages closely related to the U288 clade. Major evolutionary events (arrows) of the acquisition of plasmids and AMR genes, and the accumulation of hypothetically disrupted coding sequences (HDCSs) resulting in possible pseudogene formation are indicated in boxes.

In order to investigate the temporal relationship between the emergence of the U288 epidemic in UK pigs around the year 2000 and the acquisition of AMR genes and genome degradation, we investigated the accumulation of SNPs on ancestral lineages and constructed a time scaled phylogenetic tree from variation in the core genome of U288 clade and genetically closely related isolates. To enhance the accuracy for the determination of the molecular clock rate, we supplemented the 150 U288 and closely related strains (green and red lineages, Fig. 2) spanning the years 1948 to 2015 with 84 additional WGS of U288 strains isolated between 2006 and 2017. A maximum likelihood phylogenetic tree rooted with *S*. Typhimurium strain SL1344 outgroup was constructed from recombination-purged SNPs in the core genome. Root-to-tip accumulation of SNPs exhibited a molecular clock signal with a statistically significant fit to a linear regression model (R_2_ = 0.43, p < 0.0001) (Fig. 5A). A time dated tree was estimated in a Bayesian inference framework in order to determine the date of all nodes of the tree (Fig. 5B). This analysis predicted the most recent common ancestor (MRCA) of all isolates at approximately the year 1937 (range 1915–1957), eleven years prior to the isolation of strain LT2 in London, and the MRCA of the U288 epidemic clade in 1988 (range 1982–1994). The disruption of the *pncA* gene was likely to have occurred between 1968 and 1979. Disruption of *ybaO*, *sadA*, *yqaA* and *ygbE* along with acquisition of pU288-2 between 1980 and 1990. Disruption of the *tsr* gene and the acquisition of *cmlA*, *sul3*, *dfrA12, aadA2* and *bla*_TEM_ on mobile genetic elements on plasmid pU288-1 was likely between 1993 and 1995. Disruption of the *rocR* gene that only affected a subclade of U288 containing the reference strain S01960-05, likely occurred between 2000 and 2003.

### *S*. Typhimurium U288 isolates have a longer doubling time and exhibit greater sensitivity to desiccation compared to ST34

**Fig. 6:**
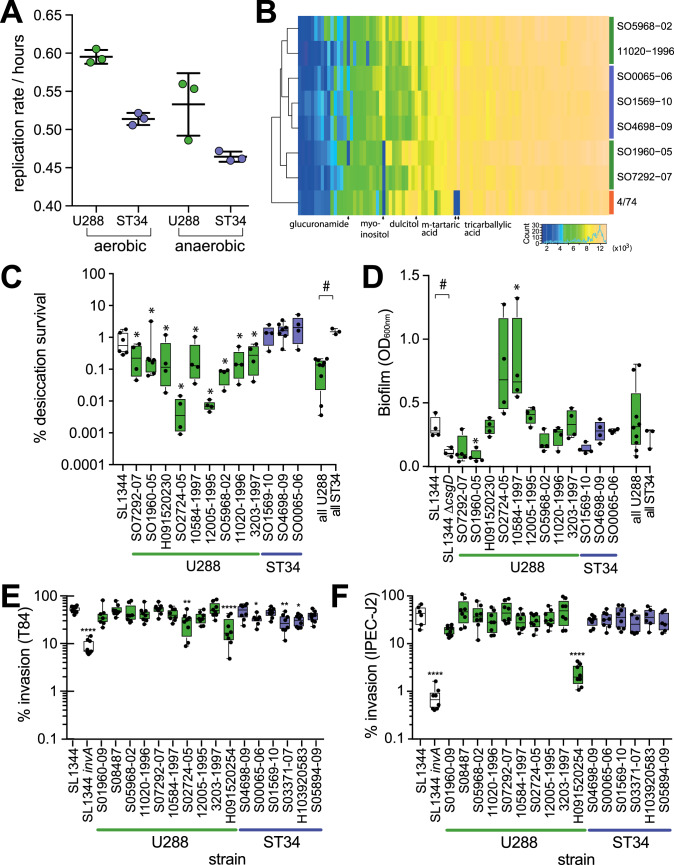
In vitro replication, carbon metabolism, sensitivity to desiccation and biofilm formation of *S*. Typhimurium U288 and ST34 isolates. **A** Circles indicate the mean doubling time of three *S*. Typhimurium U288 strains (S01960-05, S07292-07 and H09152-0230, green) and three ST34 strains (S04698-09, S00065-06 and S01569-10, blue) are indicated with the mean (horizontal bar)±with the standard error. **B** Metabolism measured using the BIOLOG phenotyping microarray platform in the presence of 95 carbon sources. The mean absorbance of at least two technical replicates were used to determine the area under the curve for each metabolite and are presented as a heat map with carbon sources in columns and eight *S*. Typhimurium strains, U288 (green bars), ST34 (blue bars) and strain 4/74 (orange bar) in rows. Unsupervised clustering of the metabolic activity of each strain and for each metabolite among test strains are indicated (left and above, respectively). **C** Proportion of CFU surviving desiccation for 24 h with reference to the initial inoculum. The mean (horizontal line), interquartile (box), and range (vertical lines) are indicated. * Indicates mean survival that were significantly different from S04698-09, and # significantly different for strains compared as indicated by brackets, assessed using a Mann-Whitney U test of significance (p < 0.05). **D** Biofilm formation estimated from the measurement of biomass by crystal violet staining of nine U288 and three ST34 strains with strain SL1344 and SL1344 Δ*csgD*::*aph* that lacks a component of curli fimbriae involved in biofilm formation as positive and negative controls, respectively. Crystal violet retention measured by absorbance at 340 nm with the mean (horizontal line), interquartile (box), and range (vertical lines) are indicated. * Indicates mean optical density that was significantly different from S04698-09, and ^#^ indicates mean optical density that was significantly different for strains compared indicated by brackets, assessed using a Mann-Whitney U test of significance (*p* < 0.05). **E**, **F** Gentamicin protection assay to estimate the invasion of T84 (**E**) and IPEC-J2 (**F**) epithelial cells by ten U288 and six ST34 strains with strain SL1344 and SL1344 Δ*invA*::*aph* that lacks a key component of the invasion associated type III secretion system 1 of *Salmonella* as positive and negative controls, respectively. The mean (horizontal line), interquartile (box), range (vertical lines) and individual data points (circles) are indicated. * Indicates mean invasion that were significantly different from S04698-09, in a Mann-Whitney U test of significance (p < 0.05). The mean and standard error of representative data from two independent biological replicates are shown.

We next compared U288 and ST34 isolates replication rate, motility, biofilm formation and ability to survive desiccation since these characteristics may be important for survival in the food chain. Strains from the U288 clade exhibited a longer aerobic and anaerobic doubling time and increased sensitivity to desiccation, but similar motility and capacity to form biofilm, compared to monophasic *S*. Typhimurium ST34 isolates. The mean doubling time for three U288 isolates was 0.6 h and 0.54 h in aerobic and anaerobic environments, respectively, compared to 0.52 h and 0.47 h for three ST34 isolates (Fig. 6A).

Since strains of each clade exhibited distinct replication rates, we compared respiration for three isolates of ST34 and four isolates of U288, utilizing a range of substrates as the sole carbon source for metabolism using the BIOLOG phenotyping microarray. All of the strains tested were able to use the majority of 95 carbon sources tested, but there was variation among approximately a quarter of substrates (Fig. 6B). The pattern of carbon source utilisation of U288 and ST34 isolates was distinct from the commonly used lab strains *S*. Typhimurium 4/74 (histidine prototroph variant of strain SL1344). The inability or diminished ability of strain 4/74 to utilise m-Tartaric, Tricarballylic acid and D-xylose was a major factor that distinguished this strain from U288 and ST34 strains. Three ST34 strains clustered together, but the U288 isolates exhibited considerably greater diversity in carbon source utilization. Utilization of myo-inositol as a sole carbon source was the most pronounced phenotype that distinguished the two clusters of U288 isolates. Of note, strain S05968-02 and 11020-1996 that were able to use myo-inositol were isolated earlier and were more deeply rooted than strains S01960-09 and S07292-07 that were unable to use this source of carbon.

We observed a clade-specific variation in tolerance to desiccation by comparing ten U288 strains and three ST34 strains. Following desiccation for 24 hours, approximately 2% of the initial inoculum remained viable for all three ST34 strains (Fig. 6C). In comparison the mean viability of U288 strains was 0.1%, but varied between 0.0001% and 0.3% among the ten U288 strains tested.

The loss of ability to form biofilm is a common feature of some host-adapted variants of *Salmonella enterica*^37,38^ (Fig. 6D). *S*. Typhimurium strain SL1344 formed moderate biofilm that was dependent on expression of the *csgD* gene as previously described.^37^ The mean biofilm formation for ten U288 strains was not significantly different from that of three ST34 strains. However, considerable variation was observed especially for the U288 strains, and two strains of U288 had a statistically significant difference in biofilm formation compared to ST34 strain S04698-09, with U288 strain S01960-05 produced significantly less biomass and strain 10584-1997 significantly greater biomass.

### *S*. Typhimurium U288 and monophasic *S*. Typhimurium ST34 isolates exhibit distinct interactions with the host

We initially evaluated the interaction of representative strains of ST34 and U288 with tissue culture cells. No difference in the ability of U288 or ST34 isolates to invade human (T84) or porcine (IPEC-J2) epithelial cells in culture was observed (Fig. 6E, F). Although, several strains of U288 and ST34 had a small but significantly lower invasion compared to strain SL1344 in T84 cells, and two U288 isolates (S01960-09 and H091520254) exhibited decreased invasion of IPEC-J2 cells.

In an initial experiment using the streptomycin pre-treated C57bl/6 mouse model of colitis we found that mice infected with randomly selected strains of the same phylogroup (ST34 or U288) exhibited similar colonisation level (Supplementary Fig. 4A), induction of Cxcl1 (KC) and Nos-2 (iNOS) (Supplementary Fig. 4B) and intestinal pathology (Supplementary Fig. 4C). However, in each case, U288 strains (i) colonised the mouse caecum to a greater level, (ii) induced higher levels of CXCL1 and NOS2 transcripts, and iii) triggered a more severe pathology, compared to that resulting from infection with ST34 strains.

**Fig. 7:**
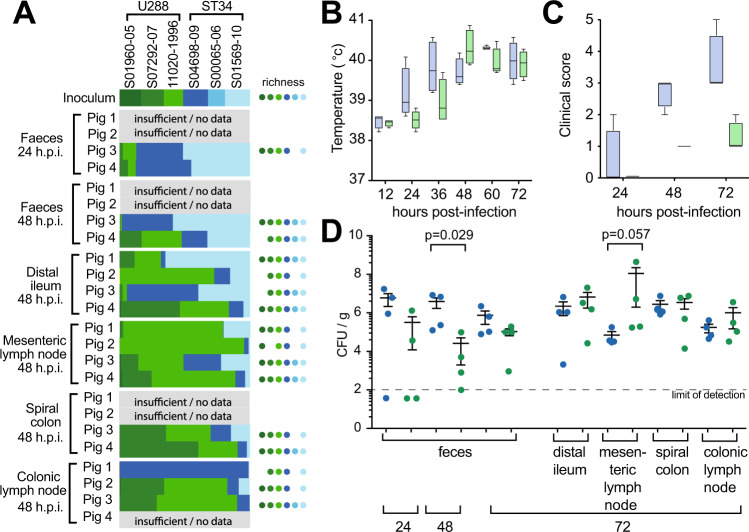
Colonisation and clinical signs of disease following oral inoculation of pigs with S. Typhimurium U288 and ST34. **A** Four pigs were challenged orally with an inoculum containing six wild-type independently tagged strains (WITS) of *S*. Typhimurium (three DT193 and three U288) in approximately equal proportions. Whole genome sequence of the population of the WITS in the inoculum and recovered from infected pigs was used to enumerate each strain based on the unique sequence tag. The relative abundance of each strain is denoted by bars and the number of strains in each sample (richness) is indicated by circles based on the colour code indicated. Separately, four pigs were challenged orally with S04698-09 (ST34), or 11020-1996 (U288) strains to investigate the signs of disease and colonisation. **B**–**D** Groups of four pigs were inoculated with approximately 1 × 10^10^ colony forming units of either ST34 S04698-09 (blue bars or circles) or U288 11020-1996 (green bars or circles). **B** Rectal temperatures of the pigs were monitored over 72 h of infection and **C** Clinical scores derived from physiological signs and faecal consistency are box plots showing the median interquartile range. **D** The mean and standard error of viable counts of bacteria shed in the faeces at 24, 48 and 72 h post-inoculation and tissues collected 72 h post-inoculation.

To compare the ability of six strains to colonise pigs, the reference strains of U288 (S01960-05) and ST34 (S04698-09) and two additional randomly selected strains of U288 (S07292-07 and 11020-1996) and ST34 (S00065-06 and S01569-10) were modified by insertion of unique sequence in the chromosome to facilitate identification by sequencing, and four pigs were inoculated orally with an equal mixture of all six isolates. Colonisation was investigated after 48 hours by sequencing cultured homogenates of faeces and tissue and enumeration of the sequence reads for the strain-specific tags. U288 and ST34 exhibited a distinct pattern of colonisation (Fig. 7A). ST34 isolates S04698-09 and S01569-10, were more abundant in three of four faeces samples 24 and 48 h post inoculation, compared to U288 strains S07292-07 and 11020-1996. Isolates of neither variant were consistently dominant in the distal Ileum. In contrast, the U288 isolates were generally more abundant in the mesenteric lymph nodes and the tissue of the spiral colon.

To further investigate the colonisation of pigs after oral inoculation, groups of four pigs were inoculated with either U288 strain S011020-1996 or ST34 strain S04698-09 in single infection experiments (Fig. 7B–D). Rectal temperatures and clinical scores were recorded for all pigs throughout the infection, and at 72 h post-inoculation the pigs were sacrificed, and colonisation of the faeces and tissues was determined. Pigs inoculated with the U288 strain had significantly lower body temperatures at early time points post-inoculation (Fig. 7B), and this was reflected in significantly lower clinical scores than those inoculated with the ST34 strain (Fig. 7C). Colonisation by both the U288 and ST34 isolates was consistent with the mixed inoculum experimental infections. The mean *Salmonella* CFU present in faeces was also lower at all time points in pigs inoculated with U288, compared with ST34 (Fig. 7D). At 48 h post-inoculation approximately 100-fold more ST34 than U288 were present in the faeces. Similar colonisation for each isolate was observed in the distal ileum and spiral colon, but U288 was present in higher numbers in the mesenteric and colonic lymph nodes (Fig. 7D), consistent with the mixed inoculum experiments.

## Discussion

Estimation of relative risk of human infection based on phage-typing is potentially misleading, due to potential polyphyletic clusters of common phage-types. Our phylogenetic analysis indicated that the majority of U288 isolates are from a single clonal group, but that this clade also contained a number of isolates that were not identifiable by phage typing as U288, therefore, the contribution to human infection may be underestimated. Conversely, a proportion of the *S*. Typhimurium U288 isolates from human clinical infections were only distantly related to the clonal group of *S*. Typhimurium U288 associated with pigs in the UK, and therefore, contribute to an over estimation of the pig associated U288 genotype to human infection. We more accurately estimated the relative contribution of the U288 clade variant and monophasic *S*. Typhimurium ST34 variant to human infection by determining the relative contribution of the *S*. Typhimurium U288 clade isolates and monophasic *S*. Typhimurium ST34 clade isolates to human clinical infections in the UK between April 2014 and December 2015. Of 1826 *S*. Typhimurium isolated in this period 33 (1.9%) were from the U288 clade. In contrast, 894 isolates (54%) were from the monophasic *S*. Typhimurium ST34 clade. Whole genome sequence or sequence polymorphisms specific to the U288 clade have the potential to improve surveillance to identify the U288 pathovariant in pig herds.

The majority of *S*. Typhimurium U288 isolates from livestock and human infections were present in a phylogenetic clade that evolved from a common ancestor that was closely related to strain LT2. LT2 was isolated at Stoke Mandeville hospital in 1948 and has been used in a large number of studies on the genetics and biochemistry of *S*. Typhimurium.^39^ Approximately 50 years elapsed since the isolation of LT2 before U288 was first detected by epidemiological surveillance in the UK pig population, around the year 2003.^8^ The zoonotic source of the infection caused by the LT2 strain and that of the common ancestor with U288, is not known.^39^ Evolution of successive descendants of the LT2-like hypothetical ancestor gave rise to multiple lineages, including one that gave rise to the U288 epidemic clade during the 1980s. Evolution of U288 was characterized by the step-wise acquisition of genes involved in resistance to multiple antibiotics, and the accumulation of genome sequence polymorphisms, some of which resulted in interruption of coding sequences. Evolution culminated with the acquisition of AMR genes on pU288-1 and disrupted of the *tsr* gene around 1995, the date of the common ancestor of the majority of the isolates in the U288 clade. The U288 clade may therefore have been evolving in the pig population before its first detection and rapid spread around the year 2003. The slow emergence of the U288 clade may have been associated with a gradual adaptation to a unique niche in the pig population, but it may also reflect a lag in time from emergence to detection by surveillance, as was proposed for other epidemic serotypes such as *S*. Enteritidis in poultry layer flocks around 1980, after the eradication of *S*. Gallinarum more than a decade previously.^40^

The acquisition of resistance to antimicrobials is a key factor in the emergence of bacterial pathogens over the past 50 years, and is often the evolutionary event immediately preceding the spread and clonal expansion of a new clone.^2,41^ A U288 isolate was previously reported to encode antimicrobial resistance genes on a pSLT-like plasmid pU288-1 (*dfrA12, aadA2, cmlA, aadA1, sul3* and *bla*_TEM_, and *sul2*, *strA*, *strB*, *tetA*(A) and *cat*) on an IncQ plasmid pU288-2.^35^ The pU288-2 plasmid was likely acquired first as it is present in isolates from throughout the U288 clade. Insertions carrying AMR genes in pU288-1 may have occurred later than the acquisition of pU288-2, since a group of seven U288 isolates that form a more deeply rooted, basal clade, lacked the pU288-1-associated AMR genes. The majority of the U288 isolates in our analysis were direct descendants of the hypothetical ancestor that acquired AMR genes on pU288-1, and few from that of the basal clade that lacked these genes, suggesting pU288-1 evolution was an important event in the success of the U288 epidemic clade. However, despite a number of examples of apparent loss of AMR genes associated with both pU288-1 or loss of the pU288-2 plasmid (loss of AMR genes and the IncQ replicon), there were just three isolates that had lost both concurrently, highlighting the importance of MDR.

Sequence polymorphisms in *S*. Typhimurium U288 strain S01960-05 resulted in disrupted coding sequences affecting 26 genes, with reference to strain SL1344, a commonly used lab strain. Sixteen of these polymorphisms were predicted to have occurred before the hypothetical LT2-like ancestor of the U288 and related clades (red and green lineages in Fig. 2). Five coding sequences (SL0337, *assT3*, *yhbE*, *cutF* and *yciW*) were disrupted in all descendants of the hypothetical LT2-like ancestor, and one other (*oadA*) in all but one deeply rooted lineage. However, eight genes (*pncA*, *yqaA*, *ybaO sadA*, *ygbE*, *tsr*, *oatA* and *rocR*) were disrupted in either the hypothetical ancestor of all U288 clade isolates, or subsequently in a subset of descendant lineages, in a stepwise manner, similar to that reported previously for the *S*. Typhimurium ST313 pathovariant.^42,43^ Ancestral state reconstruction indicated that the first gene to be disrupted specifically in the U288 lineage was *pncA*, an event that coincided with the acquisition of pU288-2, encoding AMR genes. Disruption of *pncA* is therefore, characteristic of the U288 clade. PncA is a nicotinamidase, a component of one of the pyridine nucleotide cycle (PNC) pathways, involved in the recycling nicotinamide adenine dinucleotide (NAD). The PncA dependent PNC pathway is probably more active in scavenging of pyridine compounds present in the environment.^44^ NAD is central to metabolism in all living systems, participating in over 300 enzymatic oxidation-reduction reactions,^44^ and therefore, conditions where de novo synthesis of NAD is limited by the availability of tryptophan or aspartate, the inability to use exogenous pyridines may limit metabolism. Perhaps significantly, the *pncA* gene is also disrupted in *S*. Choleraesuis, a serotype that is highly host-adapted to pigs and replicates more slowly than *S*. Typhimurium in the pig intestinal mucosa.^45^ Chronologically, the next events were the disruption of the *yqaA*, *ybaA*, *sadA* and *ygbE* genes. SadA is a surface localised adhesin that contributes to cell-cell interactions and therefore multicellular behaviour,^46^ while the function of *yqaA*, *ybaA and ygbE* are unknown. Disruption of *tsr*, that encodes a methyl accepting chemotaxis protein involved in energy taxis and colonisation of Peyer’s patches in the murine model of infection, was acquired by a common ancestor of the majority of the U288 clade. This polymorphism occurred on an internal branch of the tree that coincided with the acquisition AMR genes inserted on pU288-1 and a clone that spread successfully through the pig population. Insight into the role of genes present as HDCS in U288 comes from a functional genome screen of genes required for colonisation of the pig colon using a transposon insertion library.^47^ Transposon insertions in *yqaA*, *ybaA, ygbE* had no affect on colon colonisation and insertions in *pncA* and *tsr* were not present in the transposon insertion library and so their role is not known. The potential role of *sadA* is unclear from the study since just one of eight insertion mutants was recovered in significantly lower numbers from the colon of pigs, while the remaining *sadA* mutants were recovered in similar proportions to those present in the inoculum. However, several additional genes disrupted in U288 but intact had ST34 were implicated in colonisation of the pig colon in the study. In particular, several insertion mutants in *assT3* and *assT5* were recovered in significantly lower numbers indicated by a fitness score of −2 and −5.76, and may therefore contribute to the reduced intestinal colonisation of U288 isolates.

U288 and ST34 exhibited important differences in the way they interacted with the non-host environment, that could affect the likelihood that it survives in food and is transmitted on to consumers. First, considerably more viable ST34 bacteria were recovered following desiccation for 24 h, compared to U288. Many foodborne disease outbreaks due to *Salmonella* have been traced back to low moisture, ready-to-eat (RTE) foods,^48^ including dried pork products.^49^ Resistance to desiccation may be of particular significance because low oxygen tension is associated with increased resistance to a number of secondary stressors such as pH, salt, alcohol and heat.^48^ Monophasic *S*. Typhimurium ST34 also replicated at a significantly higher rate than U288 in culture, a characteristic that may result in a higher level of contamination in food. This is important because bacteria of the family *Enterobacteriaceae* are known to replicate in food stored at 7 °C, increasing up to 10,000-fold in sausage meat in two weeks.^50^ As naturally contaminated meat samples typically contained around 10^3^ CFU/g^51^ and the infective dose of *Salmonella enterica* in humans is in the range of 10^5^ to 10^9^ CFU, considerable replication in food may be necessary for transmission along the food chain until the final consumer.

*S*. Typhimurium U288 and monophasic *S*. Typhimurium ST34 are associated with distinct risk to human health, despite circulating in the same pig population in the UK.^8^ Our data suggest that this may be due to the differences in tissue tropism and levels of each variant in the pig host that has the potential to impact the likelihood that *Salmonella* enters the food during the slaughter and butchering process. The greatest risk to *Salmonella* entering pork products is the contamination of the carcass at slaughter due to errors in the evisceration process and inadequate cleaning of polishing machines.^52^ The relative contribution of *Salmonella* present in the faeces and tissues to contamination of food is not known, but increased colonisation of *Salmonella* in the caecum due to longer time in lairage correlated with contamination of the carcass at slaughter.^53^ If the level of contamination of different organs of the pig is important for transmission of U288 and ST34 through the food chain, our data is consistent with a greater role for faecal contamination of the final food product. Mean viable counts of a U288 isolate were up to two orders of magnitude lower in the faeces, but 10-fold greater in mesenteric lymph nodes, compared to an ST34 isolate. The reason for the difference in colonisation of pigs is not known. However, ST34 lack the pSLT virulence plasmid, encoding the *spv* locus that is required for invasive disease in mice and severe gastroenteritis in cattle.^54–56^ Polymorphisms in some U288 strains also affect this locus. Acquisition of the IS*26* element appears to have been accompanied by deletion of the *spvR* and *spvA* genes, also likely affecting expression of *spvB* and *spvC* as they are under transcriptional control by SpvR. Further analysis of the impact of the genotypic variation of pig colonisation of U288 and ST34 will reveal the key determinants.

Taken together, our data contribute to a better understanding of the evolutionary history and phenotypes associated with the emergence of a new *Salmonella* pathovariant. In the case of *S*. Typhimurium U288, adaptation in pigs appears to have been accompanied by a decreased risk to food safety for the consumption of pork or cross-contaminated food products by the human population. However, the consequences to the health and productivity of pigs as a result of a more invasive disease are not known but may be an important consideration for the pork production industry.

## Methods

### Bacterial strains and culture

*Salmonella* Typhimurium U288 and ST34 isolates used in this study were isolated from human clinical infections during routine diagnostic testing by Public Health England (PHE), or from animals during routine surveillance or epidemiological investigation by Animal and Plant Health Agency (APHA) (Supplementary Data 1). All sequence from samples taken from clinical infections were published previously^14,57^ and informed consent was not required as part of this study. All sequence data generated in this study are available in the SRA database under BioProject accession number PRJNA641292. In total we analysed the whole genome sequence of 2085 *S*. Typhimurium strains (Supplementary Data 1) isolated during routine surveillance by APHA and PHE. These included 166 U288 pig strains isolated by APHA composed of 89 from 2014-2015 and an additional 77 from 2005-2016 included to facilitate ancestral state reconstruction and calculation of the molecular clock rate. Additional strains isolated from animals by the APHA were included along with a collection of well-characterized *S*. Typhimurium isolates described previously^2,14^ to provide phylogenetic context. *S*. Typhimurium isolated from human clinical infections during PHE diagnostics and surveillance described previously were also investigated.^57^ Bacterial isolates were stored at −80 °C in 25% glycerol and routinely cultured overnight in 5 mL LB broth at 37 °C with shaking at 200 rpm, or on solid medium consisting of Luria Bertani (LB) broth or MacConkey containing 5% Agar, and supplemented with chloramphenicol (30 mg/l) or kanamycin (50 mg/l) as appropriate.

### Preparation of genomic DNA and sequencing of bacterial isolates

Genomic DNA for short-read sequencing reported in this study was extracted using Wizard® Genomic DNA Purification (Promega) from a culture inoculated from a single colony and incubated for 18 hours at 37 °C. Low Input Transposase Enabled (LITE) Illumina libraries were constructed using a modified protocol based on the Illumina Nextera kit (Illumina, California USA). A total of 1 ng of DNA was combined with 0.9 µl of Nextera reaction buffer and 0.1 µl Nextera enzyme in a reaction volume of 5 µl and incubated for 10 minutes at 55 °C. To this 5 µl mixture containing the DNA we added 2.5 µl of 2 µM custom barcoded P5 and P7 compatible primers, 5 µl 5x Kapa Robust 2 G reaction buffer, 0.5 µl 10 mM dNTPs, 0.1 µl Kapa Robust 2 G enzyme and 10.4 µl water were mixed and DNA amplified by incubating the sample at 72 °C for 3 minutes, followed by 14 PCR cycles consisting of 95 °C for 1 minute, 65 °C for 20 seconds and 72 °C for 3 minutes. 20 µl of amplified DNA was added to 20 µl of Kapa beads and incubated at room temperature for 5 minutes to precipitate DNA molecules >200 bp onto the beads. The beads were then pelleted on a magnetic particle concentrator (MPC), the supernatant removed, and two 70% ethanol washes performed. Beads were left to dry for 5 minutes at room temperature before being re-suspended in 20 µl of 10 mM Tris-HCl, pH8. This was then incubated at room temperature for 5 minutes to elute the DNA molecules. Beads were harvested with an MPC and the aqueous phase containing the size selected DNA molecules transferred to a new tube. The size distribution of each purified library was determined on a PerkinElmer GX by diluting 3 µl of the size-selected library in 18 µl 10 mM Tris-HCl, pH8. Equimolar pool purified libraries were then subjected to size selection on a Sage Science 1.5% BluePippin cassette recovering molecules between 400 and 600 bp. QC of the size selected pool was performed by running 1 µl aliquots on a Life Technologies Qubit high sensitivity assay and an Agilent DNA High Sense BioAnalyser chip and the concentration of viable library molecules measured using qPCR. 10 pM library pools were loaded on a HiSeq4000 (Illumina, California, USA) based on an average of the qubit and qPCR concentrations using a mean molecule size of 425 bp.

### Phylogenetic reconstruction and time-scaled inference

Paired-end raw sequence data for each isolate were mapped to the SL1344 reference genome (FQ312003)^58^ or S01960-05 (PRJEB34597)^12^ using SNIPPY (version 3.0) (https://github.com/tseemann/snippy). The size of the core genome was determined using snp-sites (version 2.3.3),^59^ outputting monomorphic as well as variant sites and only sites containing A,C,T or G. A multifasta alignment of variant sites was used to generate a maximum likelihood phylogenetic tree with RAxML using the GTRCAT model implemented with an extended majority-rule consensus tree criterion.^60^ The genome sequence of *S*. Heidelberg strain SL476 (NC_011083.1) was used as an outgroup in the analysis to identify the root and common ancestor of all *S*. Typhimurium strains. To identify *S*. Typhimurium genomes in the Enterobase database in the same hierarchical clustering level as the main U288 clade^34^ we determined the lowest genetic difference cluster level (HCC20-201) that contained all isolates that were reported as U288 (accessed December 2020). The relationship of core genome sequence types was visualised using grapetree implemented within Enterobase.^61^

To infer the time of nodes on the phylogeny, we used the BactDating software package implemented in R,^62^ sequence variation in the core genome with recombination purged using Gubbins with five iterations.^63^ The resulting sequence alignments were used to construct a maximum likelihood phylogenetic tree using RAxML rooted on *S*. Typhimurium SL1344 genome as the outgroup. The Markov chain Monte Carlo was run for 1 million iterations and the convergence and mixing of chains were 113.3, 129.5, 145.8 (for μ, σ, α, respectively) calculated using the R package corda.^64^

### Pangenome analysis and in silico genotyping

The pangenome of 132 U288-clade, 90 ST34-clade and 114 additional *S*. Typhimurium from a representative collection described^14^ (Supplementary Data 1) was determined using Roary software.^12^ The presence of antibiotic resistance, virulence and plasmid replicon genes in short-read data was determined by mapping short read sequence data to databases ResFinder,^65^ VFDB^66^ and PlasmidFinder^67^ of candidate genes and local assembly using Ariba with a 90% minimum alignment identity.^68^ This tool was also used to determine the presence of specific genes or gene allelic variants. The results of the ARIBA determination of the presence or absence of specific genes were confirmed using SRST2^69^ setting each alternative form of the gene as a potential allele. SRST2 was also used to verify the ARIBA findings of the VFDB data set, as the presence of orthologous genes in the genome was found to confound the interpretation of results.

### Construction of wild type isogenic tagged strains (WITS) and single knock out

A modified recombineering method based on the Lambda Red system was used to construct knockout mutations in *S*. Typhimurium SL1344 and WITS.^70^ WITS were constructed to provide a kanamycin resistance selectable marker (*aphI*) and unique sequence tag inserted in the genome to distinguish and quantify pooled populations of strains in mixed inoculum experiments of pigs by sequencing. Briefly, primers where used to amplify the *aphI* gene from pKD4 and recombination into the genome was directed by the inclusion of 50 nucleotide sequence flanking the insertion site (Supplementary Table 1). For the construction of WITS of S04698-09, S00065-06, S01569-10, S01960-05, S07292-07 and 11020-1996 WITS, insertion was directed to the intergenic region of *iciA* and *yggE* at orthologous position 3,247,245 in SL1344.^58^

### Determination of growth rate, biofilm formation and desiccation survival

For determination of growth rate of randomly selected U288 strains (S01960-09, S07292-07 and H09152-0230), and ST34 strains (S04698-09, S00065-06 and S01569-10), bacterial cultures in LB broth were diluted to approximately 1 × 10^5^ CFU per ml and incubated at 37 °C in aerobic or anaerobic (85% N_2_, 10% H_2_ and 5% CO_2_) environments and viable bacteria in colony-forming units (CFU) enumerated by serial dilution and culture on LB agar at 1, 3, 5 and 7 h post-inoculation. Doubling time was calculated in the exponential range of growth using the mean from three biological replicates. Determination of survival after desiccation of strains SL1344, S04698-09, S00065-06, S01569-10, S01960-05, H09152-0230, S02724-05, 10584-1997, 12005-1995, S05968-02, 11020-1996 and 3203-1997 was based on a method previously described.^71^ Briefly, bacteria were cultured in LB broth at 37 °C with shaking for 18 h, harvested by centrifugation, washed with phosphate-buffered saline pH7.4 (PBS) and re-suspended in PBS and adjusted to OD_600nm_ of 1. 0.05 ml of cell suspension were added to polystyrene 96 well plate (Nunc) and desiccated at 22 °C, 36% relative humidity (RH). Desiccated plates were stored in a sealed vessel containing saturated potassium acetate solution, to maintain RH at 36% and incubated at 22 °C for 24 h. Cells were re-suspended in 0.2 ml PBS and viable counts were determined by culture of serial 10-fold dilutions on LB agar. The percentage survival was calculated from at least three biological replicates. To study biofilm formation of the same strains as for desiccation, liquid cultures were incubated statically in polystyrene 96-well plates at 22 °C for 24 hours, washed once with PBS pH7.4, and attached bacteria were stained with crystal violet and absorbance read at 340 nm. Data points represent the mean of at least three biological replicates.

### Metabolic profiling using OMNIlog microarray system

To assess utilisation of carbon sources, eight strains ST4/74, S04698-09, S00065-06, S01569-10, S05968-02, S01960-05, 11020-1996 and S07292-07 were cultured on LB agar overnight at 37 °C, inoculated into IF-0 medium containing tetrazolium dye, and added to a PM-1 plate (with 95 different carbon sources), according to the manufacturer’s instructions (IBiolC). Accumulation of purple indicator dye as a consequence of redox activity was measured every 15 minutes for 48 hours. Raw absorbance data were processed using R software and the opm package.^72^ The area-under-the-curve was a metric for total respiration of the indicated carbon sources and plotted as a heatmap using R software.

### Epithelial cell invasion assays

IPEC-J2 porcine epithelial cells and T84 human epithelial cells were cultured and routinely passaged in Dulbecco Modified Eagles Medium (DMEM), containing high glucose or low glucose for each cell line, respectively. 24-well tissue culture plates were seeded with 1 × 105 cells/ml of epithelial cell lines, and incubated at 37 °C 5% CO_2_ overnight. Stationary phase cultures of *S*. Typhimurium strains SL1344, SL1344 Δ*invA*::*aph*, S04698-09, S00065-06, S01569-10, S01960-05, H091520230, S02724-05, 10584-1997, 12005-1995, S05968-02, 11020-1996 and 3203-1997 were balanced to OD_600nm_ ~1.0 in PBS and used to inoculate epithelial cells at a multiplicity of infection (MOI) of 20. Cells were incubated for 30 minutes before washing 5 times with PBS, re-suspending in DMEM + gentamicin (100 mg/l) and incubating for a further 30 minutes at 37 °C in 5% CO_2_ atmosphere to kill extracellular bacteria. The medium was then replaced with DMEM + gentamicin (10 mg/l) and the plates were incubated for 60 minutes at 37 °C in 5% CO_2_ atmosphere. Plates were then incubated again at 37 °C in 5% CO_2_ atmosphere for 90 minutes, before a final wash with PBS + 0.1% Triton-X100. Plates were left for 2 minutes and cells were disrupted by vigorous pipetting for one minute followed by serial dilution and culture on LB agar to determine viable counts.

### Streptomycin pre-treated mouse infections

Ethical approval for the experimental mouse infections was granted following review by the Animal Welfare and Ethical Review Body (University of East Anglia, Norwich, UK) under project licence PPL 70/8597. A streptomycin pretreated model of colitis was used.^43,73^ Groups of five female 6–9 week old specific pathogen free C57bl/6 mice were randomly chosen and housed together in individually ventilated cages with food and water *ad libitum*. Mice were administered 20 mg of streptomycin sulphate by oral gavage 24 h prior to inoculation of *S*. Typhimurium. Five *S*. Typhimurium strains S00065-06, S01569-10, S01960-05, 11020-1996 and S07292-07 were cultured for 18 hours in 50 ml LB broth with shaking and approximately 5 × 10^6^ CFU were inoculated orally in 0.2 ml of PBS pH7.4 by gavage. A control group of five mice were administered only the PBS buffer. PBS pH7.4 was used to mimic physiological osmolarity and pH to maintain viability. On day 3 post-inoculation mice were killed by asphyxiation with slowly rising CO_2_ concentration and then the caecum was aseptically removed. An approximately 3 mm section of the caecum halfway down the organ from the ileal junction was removed and fixed in formalin for histopathology examination of 5 μm thin sections stained with hemotoxylin and eosin. Approximately 5 mg of caecum tissue was removed and placed in RNAlater (Thermo Fisher) and stored at −80 °C. The remaining caecum (approximately two thirds) was homogenised in sterile PBS pH7.4 and serial dilutions plated on LB agar containing 0.05 mg/ml kanamycin, incubated at 37 °C for 18 h and viable counts enumerated.

### Determination of Nos2 and Cxl1 expression in mouse caecum tissue

RNA from caecum tissue was prepared using guanidinium thiocyanate-phenol-chloroform extraction with Tri Reagent (Merck). Tissue was homogenized in 1 ml of Tri reagent and disrupted using 1.4 mm zinc oxide beads in a bead beater and tissue debris removed by centrifugation. 0.2 ml of nuclease free water and 0.2 ml of chloroform were added to the supernatant, mixed and centrifuged for 15 minutes at 12,000 x g. 0.5 ml of isopropanol was added to the upper aqueous phase and centrifuged for 15 minutes at 12,000 x g. The resulting RNA pellet was washed twice with 70% ethanol, briefly dried and resuspended in 0.02 ml of RNase free water. The relative abundance of Nos2 and Cxl1 mRNA was determined by quantitative RT-PCR using primers specific to the test genes and the Gapdh house-keeping gene as the control (Supplementary Table 1) as described previously.^74^

### Mixed-strain infection of pigs

Ethical approval for experimental infections of pigs was granted following review by the Moredun Research Institute Ethical Review Committee under project licence PCD70CB48. This infection model has previously revealed differential virulence and tissue tropism of *S. enterica* serovars in pigs.^45,75^ Pigs were confirmed to be *Salmonella*-free by selective enrichment of faeces as previously described,^76^ and therefore ethical consideration precluded inclusion of negative controls. Four 6-week-old Landrace x Large White x Durock pigs were challenged orally with a mixed-strain inoculum as previously described.^76^ We tested six strains including U288 and ST34 reference strains and two additional strains of each variant that exhibited typical pathogenicity in the mouse colitis model (S01960-09, S07292-07, 111020-1996, S04698-09, S00065-06 and S01569-10) in a mixed inoculum assay. A mixed-strain inoculum was prepared by combining equal volumes of individual cultures of the six strains grown statically at 37°C for 16 hours in LB broth supplemented with 50 mg/l kanamycin, which were optical density (OD_600nm_) standardized to contain 8.9 log_10_ CFU/ml. The number of CFU in the inoculum was determined by plating 10-fold serial dilutions of the inoculum on MacConkey agar containing 50 μg/ml kanamycin. Aliquots of the inoculum were stored at −20 °C for DNA extraction. Five ml of the mixed-strain inoculum was mixed with 5 ml of antacid [5% Mg(SiO_3_)_3_, 5% NaHCO_3_, and 5% MgO in sterile distilled water] to promote colonization and administered orally by syringe before the morning feed. Pigs were fed as normal following challenge. Rectal temperatures were recorded every 24 hours and faecal samples were collected at 24 and 48 hours post-infection. The endpoint of the experiment was humane euthanasia at 72 hours post-inoculation. A section of distal ileal mucosa, mesenteric lymph nodes (MLNs) draining the distal ileal loop, a section of spiral colon, colonic lymph nodes (CLNs) and a section of liver were collected. Lymph nodes were trimmed of excess fat and fascia, and the sections of distal ileum and spiral colon were washed gently in PBS to remove nonadherent bacteria. One gram of each tissue was homogenized in 9 ml of PBS in gentleMACS M tubes using the appropriate setting on the gentleMACS dissociator (Miltenyi Biotec). Homogenates were filtered through 40-μm-pore-size filters and an aliquot was used to determine viable counts. The remaining homogenate was spread onto 10 MacConkey agar plates (500 μl per plate) containing 50 μg/ml kanamycin and incubated overnight at 37 °C. The bacterial lawns recovered from each sample were collected by washing with PBS, and the pellets were stored at −20 °C for DNA extraction. Genomic DNA (gDNA) was extracted from the pellets using the NucleoSpin tissue kit (Macherey-Nagel), according to the manufacturer’s instructions. The quality and quantity of DNA were assessed initially by NanoDrop 3300 (Thermo Scientific), and samples with an *A*_260/280_ of < =1.8 were considered suitable for library preparation. These were confirmed further by using the DNA ScreenTape (Agilent Technologies) and the Qubit double-stranded DNA (dsDNA) BR assay kit (Life Technologies), respectively. One microgram of gDNA with a DNA integrity number (DIN) of < =6 was used for library preparation using the TruSeq PCR-free library preparation kit (Illumina) according to the manufacturer’s protocol. Whole genome sequencing on the HiSeq system (Illumina) followed by bioinformatics analysis were performed as previously described,^76^ with the exception that strains were quantified by mapping sequence data to the unique WITS tag sequence incorporated chromosomally into each strain. Sequence data was submitted to the NCBI SRA database (Supplementary Table 2). For each strain, the percentage in a population was calculated as the average WITS frequency x 100. Data are presented as the mean ± standard error of the mean (SEM).

### Single-strain infection of pigs

From the strain phenotypes identified in the mixed-strain infection, one representative strain of each ST34 and U288 clade that exhibited the greatest colonisation was selected for in vivo phenotype validation, S04698-09 and 11020-1996, respectively. The strains were grown statically at 37 °C for 16 hours in LB broth supplemented with 50 mg/l kanamycin and the optical densities (OD_600nm_) were standardized to contain 9.2 log_10_ CFU/ml, which was confirmed retrospectively by plating 10-fold serial dilutions on MacConkey agar. Groups of four *Salmonella*-free pigs were challenged orally with 5 ml of each strain as described above. Rectal temperatures were recorded every 12 hours and faecal samples were collected every 24 hours post-infection. Clinical scores were calculated for each animal using their temperatures, physiological signs and faecal consistency. At 72 hours post-infection, tissue samples were collected and processed for viable counts as described above. The bacterial load of each strain in each tissue of the infected pigs was determined. Data are presented as the mean ± SEM.

### Statistics and reproducibility

Statistical tests were performed in GraphPad Prism version 8.00 (GraphPad Software). The viable counts of bacteria in each case is presented as mean ± SEM, and differences between strains were analysed using a two-sided Mann-Whitney test of significance. Area under the curve analysis followed by a two-sided Mann-Whitney test was used to analyse the cumulative clinical scores of the infected pigs during single-strain infections. *P* values of ≤0.05 were considered to be statistically significant. The exact sample size for each experimental group are detailed in the text or in the data repository at 10.6084/m9.figshare.14046635.v1.

## Supplementary information

Supplementary Information

Supplementary Data 1

Description of Additional Supplementary Files

## Data Availability

All data is freely available in publicly accessible data bases under accession numbers reported in Supplementary Data and previously reported.^14,57^ Source data for main figures can be accessed from the figshare database.^77^ Materials and all other data are available from the authors at reasonable request.
