# Supplementary material for: Ecological niche adaptation of Salmonella Typhimurium U288 is associated with altered pathogenicity and reduced zoonotic potential
Source: Commun Biol. 2021 Apr 23;4:498. doi: 10.1038/s42003-021-02013-4 (PMC8065163; doi:10.1038/s42003-021-02013-4)
Supplement: Supplementary file 1 — Supplementary Information [file 42003_2021_2013_MOESM1_ESM.pdf]

Ecological niche adaptation of a bacterial pathogen associated with reduced zoonotic potential

Supplementary Figures and Tables

Mark Kirkwood[1], Prerna Vohra[2], Matt Bawn[1,3], Gaëtan Thilliez[1], Hannah Pye[1], Jennifer Tanner[1], Cosmin Chintoan-Uta[2], Priscilla Branchu[1], Liljana Petrovska[4], Timothy Dallman[5], Neil Hall[3], Mark P. Stevens[2] and Robert A. Kingsley[1,6*]

[1] Quadram Institute Biosciences, Norwich Research Park, Norwich, UK

[2] Roslin Institute, Edinburgh, UK

[3] Earlham Institute, Norwich Research Park, Norwich, UK

[4] Animal and Plant Health Agency, Addlestone, UK

[5] Gastrointestinal Bacteria Reference Unit, National Infection Service, Public Health England, London, UK

[6] University of East Anglia, Norwich, UK

*Correspondence: rob.kingsley@quadram.ac.uk

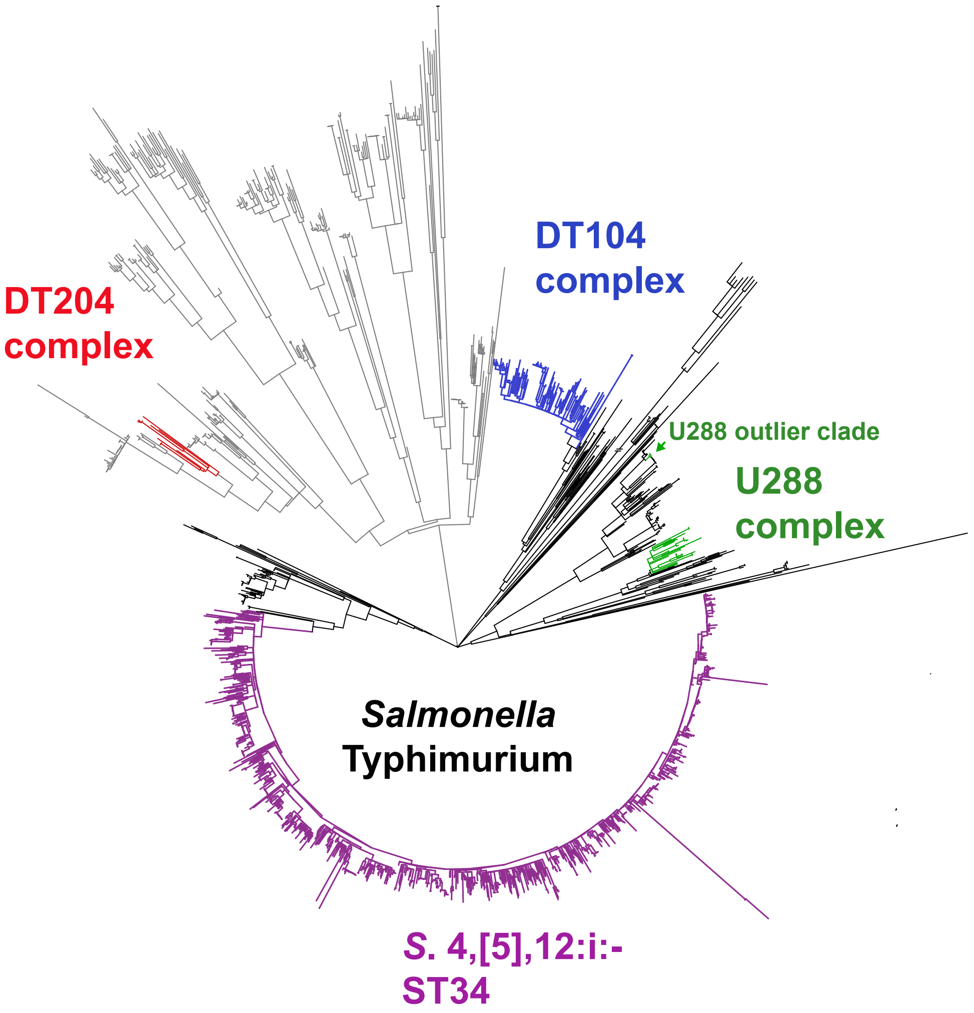

Supplementary Figure 1. Maximum likelihood estimation of the phylogeny of 1826 *S.* Typhimurium and monophasic *S.* Typhimurium isolated from human clinical infections in England and Wales in 2014-2015. Lineages comprising the U288 complex (green), ST34 (purple), DT104 complex (blue) and DT204 complex (red) clades are indicated.

A

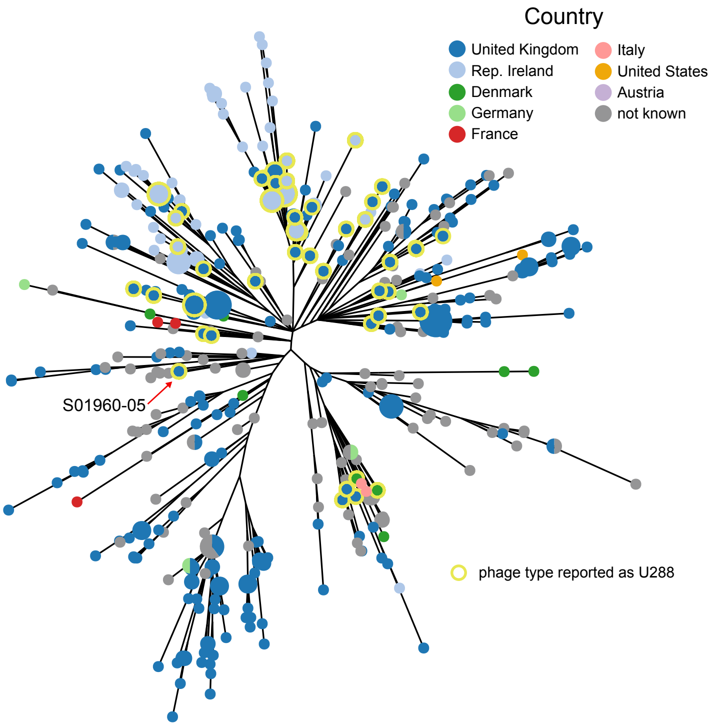

B

Country	no. HCC20-201 in enterobase	no. S. Typhimurium in enterobase	HCC20-201 (%)
Austria	1	13	8
Denmark	8	1139	0.7
France	3	1947	0.15
Germany	4	476	0.8
Ireland	71	1039	7
Italy	2	40	5
UK	254	12103	2
US	2	8802	0.02

Supplementary Figure 2. Minimum spanning tree showing the relationship of *S*. Typhimurium Enterobase hierarchical cluster HCC20-201 containing the main U288 clade. (A) Whole genome sequences with the same allelic profile are indicated by circles colour coded by the country source according to the key (inset). Where the phage type was reported as U288 (yellow ring) are indicated. (B) Table reporting the number of HCC20-201 genomes from each represented country and the percentage of *S*. Typhimurium genomes of isolates to each country in the Enterobase database that were in HCC20-201.

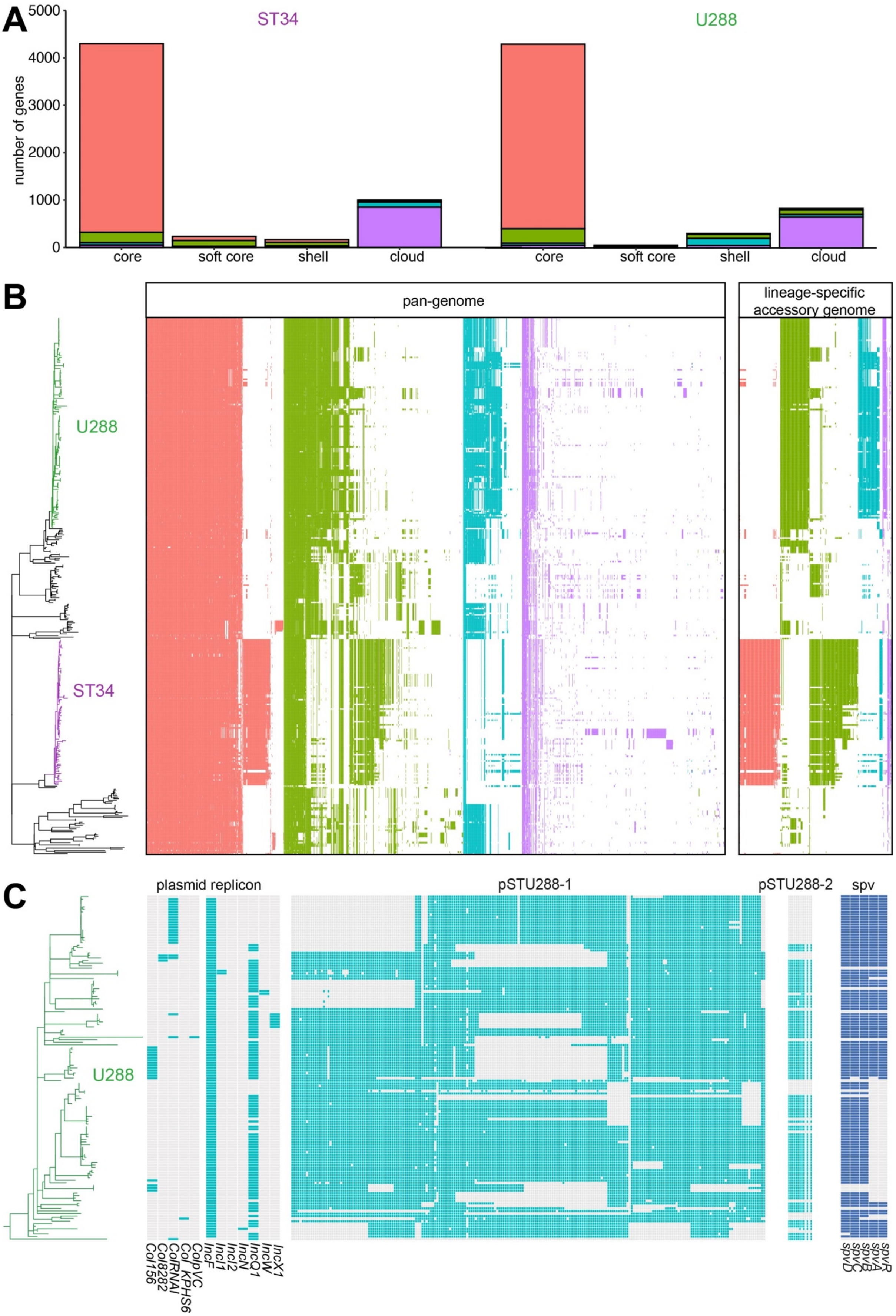
A
B
C
ST34
U288
number of genes
5000
4000
3000
2000
1000
0
core
soft core
shell
cloud
pan-genome
lineage-specific
accessory genome
plasmid replicon
pSTU288-1
pSTU288-2
spv
Col156
Col8282
ColRNAI
Col_KPHS6
ColpVC
IncF
IncI1
IncI2
IncN
IncQ1
IncW
IncX1
spvD
spvC
spvB
spvA
spvR

Supplementary Figure 3. Analysis of the pangenome of *S*. Typhimurium U288 and ST34. (A) Summary of gene families in the core, softcore, shell and cloud genomes, subdivided to indicate contribution of non-phage genes present on the chromosome (red), phage genome (green), plasmid genes (cyan) and unclassified (purple). (B) presence of gene families within *S*. Typhimurium with U288 lineages (green) and ST34 (purple) indicated. Maximum likelihood tree is the same as that in Figure 2. Filled boxes indicate a gene family (columns) colour coded for non-phage genes present on the chromosome (red), phage genome (green), plasmid genes (cyan) and unclassified (purple). (C) Plasmid replicons, plasmid genes (pU288-1 and pU288-2) and *spv* genes present in the whole genome sequence of U288 strains.

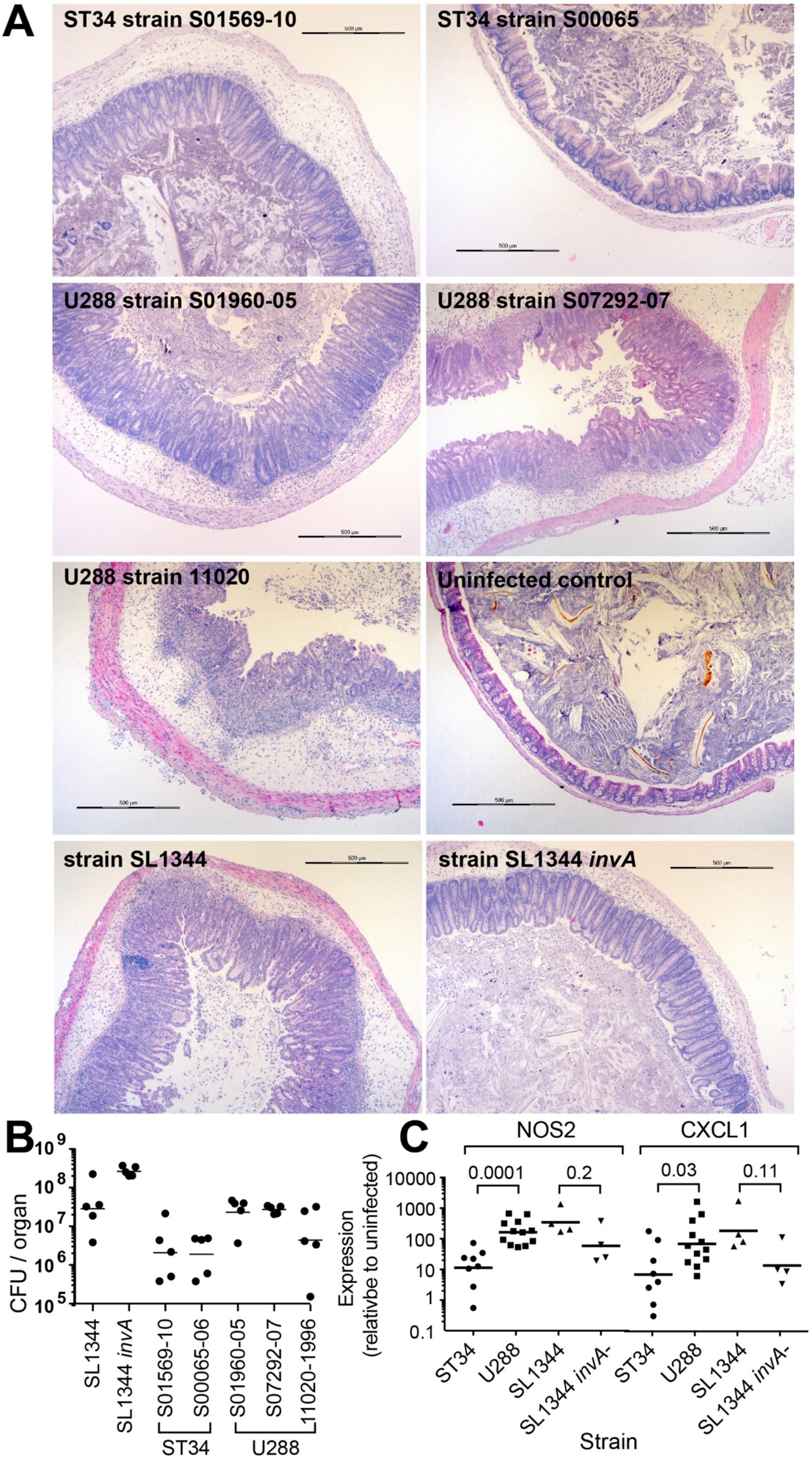
A
ST34 strain S01569-10
ST34 strain S00065
U288 strain S01960-05
U288 strain S07292-07
U288 strain 11020
Uninfected control
strain SL1344
strain SL1344 invA
500 µm
B
CFU / organ
10^9
10^8
10^7
10^6
10^5
SL1344
SL1344 invA
S01569-10
S00065-06
S01960-05
S07292-07
11020-1996
ST34
U288
C
NOS2
CXCL1
0.0001
0.2
0.03
0.11
Expression (relativbe to uninfected)
10000
1000
100
10
1
0.1
ST34
U288
SL1344
SL1344 invA-
Strain

Supplementary Figure 4. Colonisation of C57bl/6 mice and immune response following oral inoculation with *S*. Typhimurium U288 or ST34. (A) Hemotoxylin and eosin stained 5 μm sections of murine cecum from streptomycin pretreated mice infected with ST34 strains S01569-10 or S00065-06, U288 strains S01960-05, S07292-07 or 11020-1996, PBD pH7.4 uninfected control, strain SL1344 or SL1344 with a deletion in the *invA* gene. (B) Viable counts of *Salmonella* recovered from the cecum of mice on day 3 post inoculation. (C) Expression of Nos-2 and Cxcl-1 in cecal tissue of mice relative to expression in uninfected control mice determined by real-time PCR using the delta-delta Ct method. P-values using the Mann-Whitney test of significance for comparison of fold change in expression are indicated.

Supplementary Table 1. Oligonucleotide primers used in this study.

primer name	sequence 5'-3'	Notes
Primers for WITS construct		
oGT05_WITS_3	aggattagcctctctatgaatgccggatggcggcgtgaacgtcttatccgTTAGTGTAGCgtgtaggctggagctgcttcg	construction of S04698-09 WIT
oGT05_WITS_4	aggattagcctctctatgaatgccggatggcggcgtgaacgtcttatccgTCTACTGAGTgtgtaggctggagctgcttcg	construction of S01960-05
oGT05_WITS_13	aggattagcctctctatgaatgccggatggcggcgtgaacgtcttatccgGAAGTGAGACgtgtaggctggagctgcttcg	construction of S00065-06
oGT05_WITS_14	aggattagcctctctatgaatgccggatggcggcgtgaacgtcttatccgGATAGATAGCgtgtaggctggagctgcttcg	construction of S01569-10
oGT05_WITS_15	aggattagcctctctatgaatgccggatggcggcgtgaacgtcttatccgTGAGACCTATgtgtaggctggagctgcttcg	construction of S07292-07
oGT05_WITS_16	aggattagcctctctatgaatgccggatggcggcgtgaacgtcttatccgTGTAGATTCGgtgtaggctggagctgcttcg	construction of 11020-1996
oGT02_WITS	tttgtgatgaacttcaaagaagtgacgagggaataatgggtcatgcaggccatatgaatatcctccttagt	construction of all WIT strains
Primers for allelic exchange		
invAko-f	cagcgatatccaaatgttgcatagatcttttccttaattaagcccgtgtaggctggagctgcttcg	SL1344 ΔinvA::kan
invAko-r	actattgaaaagctgtcttaatttaatattaacaggatacctatacatatgaatatcctccttagt	SL1344 ΔinvA::kan
csgDko-f	agtaactctgctgctacaatccaggtcagatagcgtttcatggccgtgtaggctggagctgcttcg	SL1344 ΔcsgD::kan
csgDko-r	gggggcagctgtcagatgtgcgattaaaaaaagtggagtttcatccatatgaatatcctccttagt	SL1344 ΔcsgD::kan
primers for qRT-PCR		
Gapdh-f	TGTAGACCATGTAGTTGAGGTCA	qRT-PCR of Gapdh
Gapdh-r	AGGTCGGTGTGAACGGATTTG	qRT-PCR of Gapdh
Cxl1-f	ATGGCTGGGATTCACCTCAA	qRT-PCR of Cxl1
Cxl1-f	AGTGTGGCTATGACTTCGGTTT	qRT-PCR of Cxl1
Nos2-f	TTGGGTCTTGTTCACTCCACGG	qRT-PCR of Nos2
Nos2-r	CCTCTTTCAGGTCACTTTGGTAGG	qRT-PCR of Nos2

Supplementary Table 2. Sequence data for quantification of strains S01960-05, S07292-07, 11020-1996, S04698-09, S00065-06 and S01569-10 in the faeces, intestinal lumen and tissue of pigs.

sample type	pig number	Sample Name	Accession	BioProject
Challenge inoculum	N/A	SM0024	SAMN15596641	PRJNA641292
Ileal mucosa	1	SM0004	SAMN15596642	PRJNA641292
Ileal mucosa	2	SM0005	SAMN15596643	PRJNA641292
Ileal mucosa	3	SM0006	SAMN15596644	PRJNA641292
Ileal mucosa	4	SM0007	SAMN15596645	PRJNA641292
Mesenteric lymph node	1	SM0008	SAMN15596646	PRJNA641292
Mesenteric lymph node	2	SM0028	SAMN15596647	PRJNA641292
Mesenteric lymph node	3	SM0010	SAMN15596648	PRJNA641292
Mesenteric lymph node	4	SM0011	SAMN15596649	PRJNA641292
Colonic mucosa	3	SM0012	SAMN15596650	PRJNA641292
Colonic mucosa	4	SM0013	SAMN15596651	PRJNA641292
Colonic lymph node	2	SM0014	SAMN15596652	PRJNA641292
Colonic lymph node	3	SM0015	SAMN15596653	PRJNA641292
Colonic lymph node	4	SM0016	SAMN15596654	PRJNA641292
Faecal sample at day 1	1	SM0033	SAMN15596655	PRJNA641292
Faecal sample at day 1	2	SM0019	SAMN15596656	PRJNA641292
Faecal sample at day 2	3	SM0034	SAMN15596657	PRJNA641292
Faecal sample at day 2	4	SM0021	SAMN15596658	PRJNA641292
