# Supplementary material for: Ecological niche adaptation of Salmonella Typhimurium U288 is associated with altered pathogenicity and reduced zoonotic potential
Source: Commun Biol. 2021 Apr 23;4:498. doi: 10.1038/s42003-021-02013-4 (PMC8065163; doi:10.1038/s42003-021-02013-4)
Supplement: Supplementary file 3 — Description of Additional Supplementary Files [file 42003_2021_2013_MOESM3_ESM.pdf]

Description of Additional Supplementary Files

File name: Supplementary Data 1

Description: Whole genome sequence data analysed in this study.
